# Society Meetings

**Published:** 1901-12

**Authors:** 


### SOCIETY MEETINGS.

---

THE Buffalo Academy of Medicine held meetings during the months of October and November, 1901, as follows:

*Section on Obstetrics.*—Tuesday evening, October 29. Program: Concerning the diagnosis and treatment of the various forms of septic pelvic diseases, Fernand Henrotin, Professor of gynecology, Chicago Medical College.

*Section on Surgery.*—Tuesday evening November 5. Program: The etiology of obstruction of the bowel, with report of cases, Eugene A. Smith.

*Section on Medicine.*—Tuesday evening, November 12. Program: Some facts and fallacies concerning pulmonary tuberculosis, John H. Pryor; discussion by De Lancey Rochester and H. U. Williams.

*Stated Meeting.*—Monday evening, November 18. Program: The operation for extraction of cataract, as originally devised by Daviel. Report of case of exophthalmic goitre, with the exophthalmos limited to left eye, A. A. Hubbell.

*Section on Pathology.*—Tuesday evening, November 19. Program: The unveiling of the cell, Lewellyn F. Barker, professor of anatomy, University of Chicago.

*Section on Obstetrics.*—Tuesday evening, November 26. Program: Pelvic lesions in relation to their distinctive effects upon mental disturbances, A. T. Hobbs, London, Ont.

Reproduction of original work done in microscopic embryology, accompanied by remarks on heredity. Illustrated by stereopticon. Adele A. Gleason.

---

THE Southern Surgical and Gynecological Association held its 14th annual meeting at Richmond, Va., November 12, 13 and 14,

1901. It was well attended and the meeting was spirited. The social entertainments were of the best order. A reception was given on the evening of the first day by Dr. Hugh M. Taylor at his home, which was a delightfully appointed social function. The house was crowded to the doors with members and guests. At Dr. George Ben Johnston's luncheon, given at his residence Wednesday, it was quite the same; and at the reception given by Dr. Lewis C. Bosher at the Westmoreland Club, Wednesday evening, besides all the other delights, Polk Miller gave one of his unique and fun-provoking musical monologues relating to old plantation life. Besides these the luncheons on Tuesday and Thursday at Mrs. John L. Eubank's, at which the Doctors Williams were hosts, were most charming appointments.

Dr. William E. B. Davis, of Birmingham, was chosen president and the next meeting of the association will be held at Cincinnati.

---

THE Niagara Falls Academy of Medicine held its regular monthly meeting on Monday evening, November 4, 1901, at the residence of Dr. C. G. Leo-Wolf. A paper was read by Dr. C. E. Campbell, entitled, The correlation of life and death.

---

THE first medical congress to be held in Egypt will occur from December 10-14, 1902, under the patronage of the Khedive, at Cairo, and be devoted to three diseases of peculiar interest to the Egyptians. This is a most commendable undertaking and shows a spirit of progress which will not fail to be appreciated by the civilised nations of Europe and America. It may also be the means of stimulating further research into the causes and prophylaxis of many of the diseases which continually harass the inhabitants of the dean of nations, and may result in some concerted action looking to their complete suppression. Officers: Honorary presidents of the congress, Drs. Abbate Pacha, Pinching, and Ruffer; president of congress, Dr. Ibrahim Pacha Hassan; secretary-general of congress, Dr. Voronoff; sections: Science of medicine, Dr. Comanos Pacha, president; science of surgery, Dr. H. Milton; ophthalmology, Dr. Mohamed Bey Eloui.

---

THE Medical Society of the County of Chautauqua, will hold its semi-annual meeting at Jamestown on the second Tuesday in December, 1901, at which Dr. J. Henry Dowd, of Buffalo, will read a paper on, Tuberculosis of the Genito-urinary Tract.

